#  Neuronal Differentiation of Rat Hair Follicle Stem Cells: the Involvement of the Neuroprotective Factor Seladin-1 (DHCR24) 

**DOI:** 10.6091/ibj.1284.2014

**Published:** 2014-07

**Authors:** Samira Gilanchi, Banafshe Esmaeilzade, Akram Eidi, Mahmood Barati, Soraya Mehrabi, Fatima Moghani Ghoroghi, Maliheh Nobakht

**Affiliations:** 1 *Iran National Science Foundation, Tehran, Iran; *; 2 *Dept. of Biology, Science and Research Institute, Islamic Azad University, Tehran, Iran; *; 3 *Dept. of Anatomy, School of Medicine, Bushehr University of Medical Sciences, Bushehr, Iran; *; 4 *Dept. of Pharmaceutical Biotechnology, School of Pharmacy, Shahid Beheshti University of Medical Sciences, Tehran, Iran; *; 5 *Dept. of Neurosciences, School of New Technology, Tehran University of Sciences, Tehran, Iran; *; 6 *Dept. of Histology and Neuroscience, School of Medicine, Iran University of Medical Sciences, Tehran, Iran; *; 7 *Anti-microbial Resistance Research Center, Iran University of Medical Science, Tehran, Iran*

**Keywords:** Seladin-1 (selective Alzheimer disease indicator-1), Alzheimer disease (AD), Hair follicle stem cells

## Abstract

**Background: **The seladin-1 (selective Alzheimer disease indicator-1), also known as DHCR24, is a gene found to be down-regulated in brain region affected by Alzheimer disease (AD). Whereas, hair follicle stem cells (HFSC), which are affected in with neurogenic potential, it might to hypothesize that this multipotent cell compartment is the predominant source of seladin-1. Our aim was to evaluate seladin-1 gene expression in hair follicle stem cells. **Methods: **In this study, bulge area of male Wistar rat HFSC were cultured and then characterized with Seladin-1 immunocytochemistry and flow cytometry on days 8 to 14. Next, 9-11-day cells were evaluated for seladin-1 gene expression by real-time PCR. **Results: **Our results indicated that expression of the seladin-1 gene (DHCR24) on days 9, 10, and 11 may contribute to the development of HFSC. However, the expression of this gene on day 11 was more than day 10 and on 10^th^ day was more than day 9. Also, we assessed HFSC on day 14 and demonstrated these cells were positive for β-ш tubulin, and seladin-1 was not expressed in this day. **Conclusion:** HFSC express seladin-1 and this result demonstrates that these cells might be used to cell therapy for AD in future.

## INTRODUCTION

Alzheimer disease (AD) is a progressive neuro-degenerative disease characterized clinically by insidious onset of memory and cognition deterioration as well as daily activity impairment. The neurodegenerative process in AD may involve in β-amyloid toxicity [1].

During the past decade, many hypotheses were put forward for AD pathogenesis. Among them, the β-amyloid cascade and the tau hyperphosphorylation are the widely accepted theories. Therefore, the disease-modifying therapies mainly focus on the agents that decrease the Aβ content and tau hyperphosphorylation [2, 3].

The *seladin-1* (selective Alzheimer disease indicator-1) gene was first identified in 2000 by using a differential mRNA display approach. This approach was applied to identify the genes that were differentially expressed in selective vulnerable brain regions in AD [4], such as hippocampus, amygdala, inferior temporal cortex, and the entorhinal cortex [3].

A few years ago, a novel gene, named seladin-1 or DHCR24, was identified that was associated with neurodegeneration. In fact, this gene was found to be down-regulated in brain regions affected by AD [4, 5]. Down-regulation of seladin-1 expression in brain areas vulnerable to AD was paralleled by an increase in the amount of hyperphosphorylated tau, a protein component of neurofibrillary tangles [6].

With regard to its biological effects, seladin-1 was originally found to confer resistance against β-amyloid and oxidative stress-induced apoptosis [5, 7]. It has been also demonstrated that the ability of *seladin-1* to protect against the apoptosis elicited by oxidative stress may be related to the scavenging activity of this protein [8]. However, this ability of *seladin-1* was associated with high H_2_O_2_-scavening activity, whereas an N-terminal deletion caused loss of this activity. Furthermore, *seladin-1* effectively inhibited the activation of caspase-3, a key mediator of the apoptotic process [5, 7]. In fact, reduced seladin-1 levels prevented apoptosis in a p53-dependent manner via increased ubiquitination and degradation of p53, a protein with enzymatic activity [9].

It has been shown that the seladin-1 gene encodes 3β-hydroxysterol Δ24-reductase, which catalyzes the synthesis of cholesterol from desmosterol [10, 2]. Disruption of cholesterol homeostasis may be detrimental for cells, because cholesterol-depleted membrane would easily interact with toxic factors such as β-amyloid, the histopathological hallmark of AD [11, 12].

The seladin-1 gene (Gene Bank accession number AF261758) spans 46.4 kb, maps to chromosome 1p31.1-p33 and consists of nine exons and eight introns. This gene encodes an open reading frame of 516 amino acid residues and is located in the endoplasmic reticulum and, to a lesser extent, in the Golgi apparatus [4]. Also, *seladin-1* is the human homolog of the plant *DIMINUTO/DWARFI* gene, primarily described in *Arabidopsis thaliana* [13, 14].

Hippocampus and the sub-ventricular zone are two areas in brain that are affected in AD [2]. Interestingly, these areas are the unique regions where stem cells with a defined neurogenic potential are located in the adult brain [15]. In fact, although neural stem cells are present in many other areas of the adult brain, they do not appear to maintain the ability of differentiation into neurons [15].

Epidermal neural crest stem cells (EPI-NCSC) are multipotent stem cells derived from the embryonic neural crests which reside in the bulge of the outer root sheath of the adult murine hair follicles [16]. EPI-NCSC can be isolated as a highly pure population, generate all major neural crest derivatives, and be expanded* in vitro* into millions of cells [17, 18]. Toma* et al.* [19] reported that multipotent adult stem cells isolated from mammalian skin dermis, termed skin-derived precursors, could proliferate and differentiate into culture media and produce neurons, glia, smooth muscle cells, and adipocytes. In addition, intermediated filament protein nestin, a marker of immature and undifferentiated cell [20] was expressed in some cells in the bulge area of the hair follicle [21, 22].

In this study, we aimed to assess whether hair follicle stem cells (HFSC) could express the seladin-1 gene. Since the seladin-1 expression is reduced in AD, stem cells might be the prevalent sources of this protein.

## MATERIALS AND METHODS

***Media. ***Cells were maintained in a 95% air and 5% CO_2_ fully humidified environment at 37°C in a culture medium consisted of three parts DMEM and one part Ham’s F12 medium containing 10% fetal bovine serum, 100 U/mL penicillin, 100 μg/mL streptomycin, 0.5 µg/ml amphotericin B, 10 ng/mL epidermal growth factor (Sigma-Aldrich, CA, USA), 10^-9^M cholera toxin (Sigma-Aldrich, CA, USA), 0.5 mg/mL hydro-cortisone, and 5 µg/mL insulin [23].

***Hair follicle isolation and cultivation*** **. **The selected rats were sacrificed with ether, their heads were rinsed with 70% alcohol and disinfected with betadine, and their cheeks were then maintained. After that, the whisker area tissue was separated and incubated in 2 mg/mL collagenase I/dispase II solution (Sigma-Aldrich, CA, USA) at 37°C for 15 minutes. The connective tissue and the dermis around the follicles were removed, and the whisker follicle was lifted out. Next, the bulges were removed from the capsule and placed in collagen-coated culture flasks. They stack to the bottom of flask within one day. Three to four days after the cell adhesion, the cells started to emigrate from the bulge explants to the other sides of the flask bottom [24, 25].

***Immunocytochemistry***. Ten days after the cell culture, HFSC were plated in collagen-coated plates with 24 chambers in a growth medium overnight. The cells were then fixed in 4% paraformaldehyde for 20 minutes. Next, the fixed cells were washed three times with PBS for five minutes and incubated in a blocking buffer (10% goat serum, Sigma [CA, USA] and 0.3% Triton X-100, Fluka, CA, USA) at room temperature for 30 minutes. They were then incubated at 4°C overnight with the following primary antibodies: mouse anti-nestin monoclonal antibody (1:200, Millipore, MA, USA), mouse anti-CD34 monoclonal antibody (1:200, Sigma, CA, USA), mouse anti-KRT15 monoclonal antibody (1:1700, Sigma, CA, USA), mouse monoclonal anti-βШ tubulin antibody (1:400, Sigma-Aldrich, CA, USA), and mouse monoclonal [sL-14] to seladin-1 (1:25, Abcam, CA, USA). The next day, the cells were washed three times with PBS for five minutes to remove the unbound primary antibodies. Subsequently, they were incubated with sheep anti-mouse FITC-conjugated IgG (1-200, Sigma, CA, USA) at room temperature for 1 hour and rinsed with PBS for 3 × 5 minutes. Then, the cell nuclei were counterstained with 1 µg/mL diamino-2-phenylindole and visualized using a fluorescence microscope.

***Flow cytometry. ***After separating from the bottom of the flask, the cells were centrifuged and 1 mL of the culture media was added to the cellular plaque. The cells were then counted and divided into two groups (main and control). In the next step, the main group was centrifuged, and the following primary antibodies were added to the cellular plaque: mouse anti-nestin monoclonal antibody (1:200, Millipore, MA, USA), mouse anti-CD34 monoclonal antibody (1:200, Sigma, CA, USA), mouse anti-KRT15 monoclonal antibody (1:1700, Sigma, CA, USA), and mouse monoclonal [sL-14] to seladin-1(1:25, Abcam, CA, USA). Finally, the mixture was kept at room temperature for 1 hour. After that, 2 mL phosphate buffer was added to the main group, then it was centrifuged. Next, FITC-conjugated IgG (1:200; Sigma, CA, USA) was inserted to the cellular plaque tube, and the final mixture was incubated for 45 minutes at room temperature. Then, 2 mL of PB was added to each of the two cellular groups, and they were centrifuged. Finally, 1 mL of PB was added to the cellular plaques, and the suspension was poured into flow cytometry tubes. Absolute quantification of *DHCR24* mRNA was performed by real-time PCR (RT-PCR) based on TaqMan techno-logies, as described previously [26]. The total RNA to be subjected to reverse transcription was extracted from fetal neuroepithelial cells in the basal condition and after exposure to insulin-like growth factor 1 or high glucose concentrations. The results were referred to as microgram of total RNA, and the experiments (n = 3) were run in triplicates.

***Quantitative real time PCR for the seladin-1 transcript. ***Measurement of the seladin-1 transcript was performed by quantitative RT-PCR based on *SYBR® Green* technology (Qiagen, Germany). The total RNA was extracted from the cells previously lysed using RNX-PLUS (CinnaGen, Iran). The measurement of RNA purity was performed by a spectrophotometer, then cDNA was constructed, and the primers were prepared. The primers used for the seladin-1 gene were 

as follows: 5'CATCGTCCCACAAGTATG 3' (forward) and 3'CTCTACGTCGTCCGTCA 5' (reverse). The primers were designed using primer 3 software and then analyzed and verified using Oligo7 software. Quantitative reverse transcription was performed in three steps: 95°C for 10 minutes, 35 cycles at 95°C for 15 seconds and at 60°C for 30 seconds. Finally, the melting curve was assessed at 60-95°C. Also, the housekeeping gene, GAPDH, was used as the reference gene. Then, three experiments were performed; each of which was run in triplicates. Gene expression was evaluated by the relative approach using the relative expression software tool (REST).

***Statistical analysis. ***The data were expressed as mean ± SEM. Statistical differences were analyzed to be appropriate using student’s *t*-test. *P*<0.05 was considered statistically significant.

## RESULTS

**Fig. 1:**
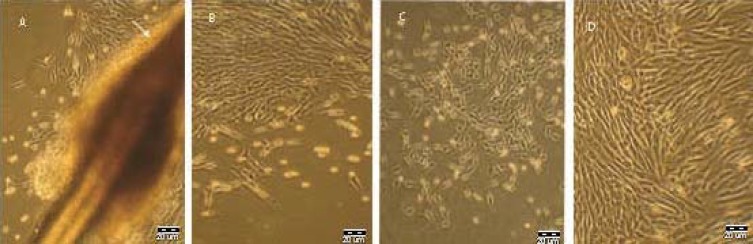
Characteristics of hair follicle stem cell (HFSC) cultures shown by inverted microscopy. The migratory cells are present on the collagen substratum. **(A)** HFSC one day after the primary culture, **(B)** migration of HFSC after the colony formation, **(C)** HFSC proliferation, and **(D)** HFSC culture after 10 days (Scale bar = 20 µm).

**Fig. 2:**
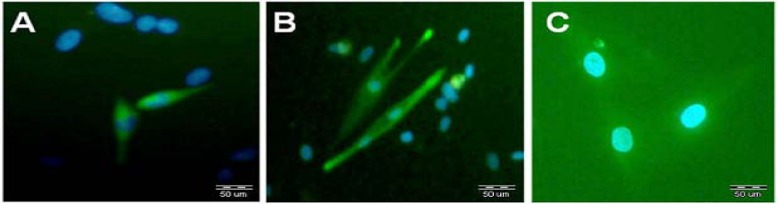
Immunocytochemical detection of nestin, CD34, and K15 antibodies in rat hair follicle stem cell. **(A)** nestin-positive cells, **(B)** CD34-positive cells, and **(C)** K15-negative cells. The nuclei were counterstained with 4',*6'-diamidino-2-phenylindole* (Scale bar = 50 µm).

**Fig. 3:**
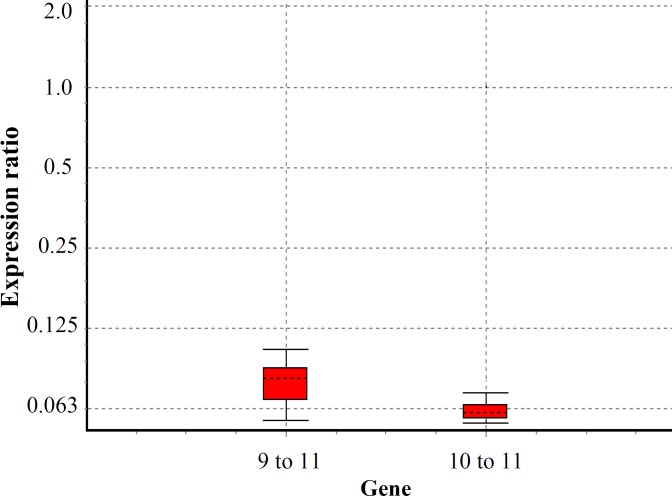
Amount of seladin-1 mRNA in hair follicle stem cells, determined by RT-PCR. The boxes represent the interquartile range. The dotted lines represent the medium gene expression. Box plots represent the minimum and maximum observations

**Fig. 4:**
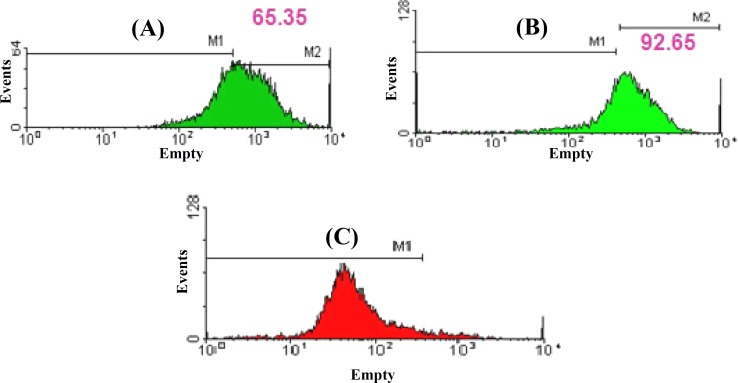
Flow cytometric analysis of the surface adhesion molecules on hair follicle stem cell (HFSC). Cultured HFSC were labeled with monoclonal antibodies specific for molecules indicated in each flow cytometric histogram. **(A)** Positive for nestin antibody, **(B)** positive for CD34 antibody, and **(C)** negative for K15

***Characterization of*** ***hair follicle stem cells*** ***.*** HFSC were successfully cultured and propagated *in vitro,* and homogeneous population of stem cells covered the bottom of the cell cultured flask after 10 days (Fig. 1). Fluorescent cell sorting at passage 1 demonstrated that the cultured cells had neural crest characteristic, because they were positive for nestin (neuron progenitor cell marker) and CD34 (stem cell specific marker) and negative for K15 (keratinocyte cell marker) antibodies (Fig. 2). The immunostaining results of 14-day HFSC with β-III tubulin (neuron-like marker) were positive, showing that EPI-NCSC can differentiate into neuron on a collagen-coated flask after 14 days (Fig. 3). After that, the flow cytometry analysis was used to assess the purity of HFSC cultures with nestin, CD34, and K15 antibodies (Fig. 4).

**Fig. 5:**
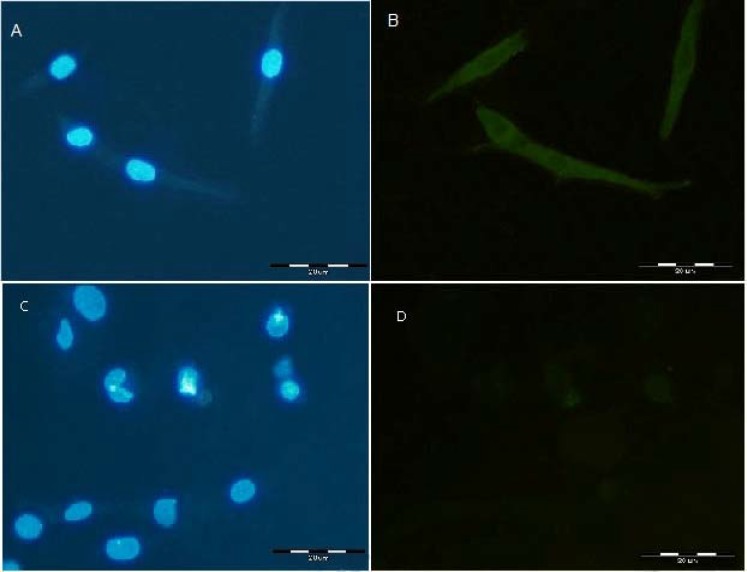
Immunocytochemical detection of the seladin-1 gene expression in rat hair follicle stem cell. **(A)** Immunostained cells with 4',*6'-diamidino-2-phenylindole* (DAPI) from the 10-day cells, **(B) **bulge cells on day 10 expressed seladin-1, **(C)** immunostained cells with DAPI from14-day cells, and **(D)** bulge cells on days 14 did not express seladin-1 (Scale bar = 20 µm).

**Fig. 6:**
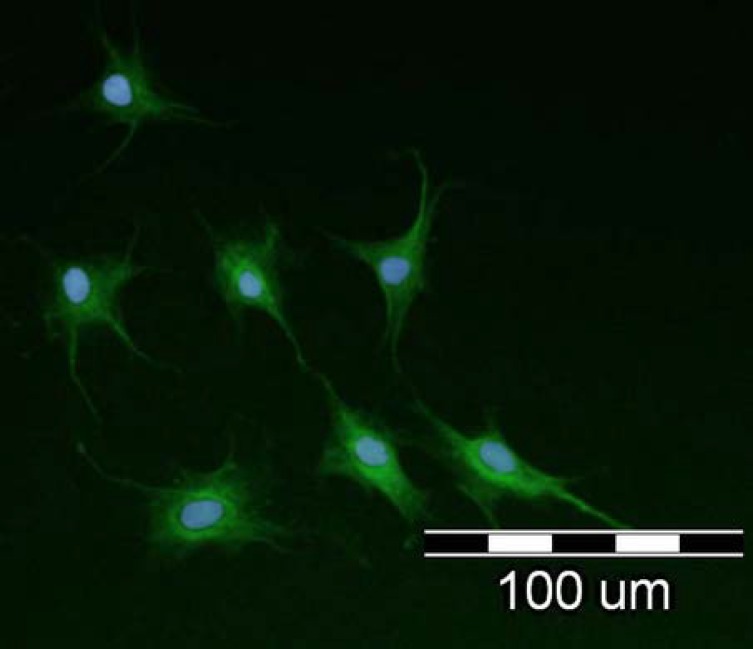
Immunocytostaining of epidermal neural crest stem cell (EPI-NCSC) of the bulge region of hair follicle stem cell after 14 days. Expression of the neuron-like marker (β-III tubulin) with EPI-NCSC. The nuclei were counterstained with 4',*6'-diamidino-2-phenylindole* (Scale bar = 100 µm).

***Assessment of the seladin-1 gene expression in*** ***hair follicle stem cells*** ***. ***The expression of seladin-1 gene was shown using immunocytochemistry (Fig. 5), and its expression level was evaluated by RT-PCR (Fig. 3). The immunocytochemistry study demonstrated that HFSC can express seladin-1, but when these cells differentiate into the neurons, they cannot express this gene (Figs. 5 and 6). In fact, after confirming the presence of this gene in 9-11-day cells with immunocytochemistry, RT-PCR was used to compare the gene expression during these three days. The results of RT-PCR showed that the seladin-1 gene had less expression in 9-day cells and extreme expression in 11-day cells. In the sample group, 9-11-day cells were different compared with the control group (*P* = 0.000). However, 10-11-day cells were down-regulated by a mean of 0.061 compared with the control group. In this group, 10-11-day cells were also different compared with the control group (*P* = 0.045).

## DISCUSSION

In the present study, we investigated the seladin*-*1 gene expression in a cell culture. The seladin-1 gene discovery as well as finding out that its expression is down-regulated in the brain areas affected by AD, the most prevalent form of late-life mental failure in humans, have opened a new window for understanding the associated neurodegenerative molecular events [4, 27].

The aim of our study was to investigate the evaluation of seladin-1 gene expression in HFSC. Possibly, the seladin-1 gene expression can be impaired in vulnerable areas to AD. In addition, neurogenic potential of some of these regions, including the hippocampus and the sub-ventricular zone of the host stem cells as well as their ability to migrate in the adult brain [15], led us to the hypothesis that this gene can also be expressed in other neuronal stem cells.

Siber-Blum *et al.* [16] described NCSC in the bulge area of the adult murine whisker follicles and introduced these cells as EPI-NCSC. According to Amoh *et al.* [28] investigation, nestin-positive K15-negative stem cells in the mouse hair follicles are multipotent and can differentiate into neurons, glial cells, keratinocytes, and other cell types. Therefore, we used HFSC of the mouse whisker hair follicles as neuronal stem cells. Furthermore, investigation of the bulge cell biology and use of bulge cells for clinical applications such as regenerative medicine or gene therapy require the isolation of living bulge cells [29]. In addition, some factors make the bulge cell a perfect stem cell; it may even be the stem cell of choice in regenerative medicine in future. For example, it is readily available from anyone, easily cultured and expanded, and does not have the ethical issues of embryonic and fetal stem cells. All of these benefits eventually led us to select this cell line for assessment of the seladin-1 gene expression [30].

The results of our study showed that the seladin-1 gene was expressed in HFSC. According to the fact that defective seladin-1 gene expression is detected in AD vulnerable brain regions, it might be linked to an impaired neuronal stem cell compartment that could be a potential risk factor for this disease [3, 4]. Based on Greeve *et al.* [4] findings, the high levels of seladin-1 gene mRNA transcription were detected in the adult hippocampus, a well-known neurogenic brain region. Our findings depicted that the seladin-1 mRNA transcription in HFSC, which are a type of NCSC, is of high importance. The finding in mice bulge cells suggested the hypothesis that stem cells are rich in bulge areas. Our results of rat hair follicle bulge area culture also supported this hypothesis [31]. Morphology-based manual microdissection was used to isolate the bulge cells from the hair follicle [32, 33]. The method of isolation was based on the migration of cells from the cultured bulge region. 

These pluripotent nestin-expressing stem cells were positive for the stem cell marker CD34 and negative for K15, suggesting that these cells are in a relatively undifferentiated state [26, 34, 35]. The nestin-expressing stem cells by immunocytochemistry and flow cytometry also indicated that the *seladin-1* gene was expressed in HFSC, when they were morphologically identical to neural stem cells, and when these cells were differentiated into neurons. As a result, we could not detect the seladin-1 gene expression, as mentioned by Benvenuti *et al.* [36]. They stated that this gene was abundantly expressed in stem cells, whereas the level of expression was markedly decreased when these cells were induced to differentiate into mature neurons. Therefore, their demonstration of seladin-1 gene was limited and variable, and when expressed, their levels were low. Therefore, we decided to measure the expression of this gene on days 9, 10, and 11 using RT-PCR. 

Interestingly, we found that the expression of this gene on day 11 was more than day 10 and on day 10 more than day 9, which was opposite to our expectation. Probably, the reason was that this gene was expressed in HFSC when the stem cell differentiated into neuron. Perhaps, the stem cells have some indices of neural cells then do not differentiate into neuron. On the other hand, this gene might be expressed in a specific age of stem cells. Presumably, its expression elevates prior to differentiation and stops after that. As observed in the 14-day cells, the expression of this gene stopped. The expression of seladin-1 gene in HFSC might be revealed in future. These cells can be used in cell therapy for AD.

In conclusion, our results indicated, for the first time, that expression of the seladin-1 gene (DHCR24) on days 9, 10, and 11 may contribute to the development of HFSC to neurons, by interfering with the trophic effects exerted by the epidermal growth factor system. It also suggested the involvement of prosurvival effect of DHCR24 as a factor. Nevertheless, additional studies, i.e. subjecting the cells to DHCR24 gene silencing factors, are required to clarify the exact role of DHCR24 in mediating the agents affecting the neuron cell differentiation.
